# Study on the health impacts of childhood asthma in China caused by air pollution

**DOI:** 10.1371/journal.pone.0338116

**Published:** 2025-12-04

**Authors:** Fangfang Ruan, Kangwei Li, Kena Mi

**Affiliations:** 1 College of International Economics and Trade, Ningbo University of Finance and Economics, Ningbo, Zhejiang, China; 2 School of Ecology and Environment, Renmin University of China, Beijing, China; 3 Economics Teaching and Research Department, The Party School of CPC Ningbo Municipal Committee, Ningbo, Zhejiang, China; Endocrinology and Metabolism Population Sciences Institute, Tehran University of Medical Sciences, IRAN, ISLAMIC REPUBLIC OF

## Abstract

Asthma is one of the major disease burdens in children. Ambient air pollution is associated with the prevalence and exacerbation of childhood asthma. Over recent decades, China has exhibited a persistent upward trajectory in pediatric asthma prevalence. This epidemiological trend necessitates a comprehensive evaluation of the health impacts associated with childhood asthma attributable to ambient air pollution exposure. This study selected PM_2.5_, NO_2_, and O_3_ as representative ambient air pollutants in China, and obtained exposure-response parameters required for health impact assessment through Meta-analysis. Then the study evaluated the health impacts of childhood asthma aged 0–14 years attributable to air pollution in 336 cities across China based on national real-time air quality monitoring data. Hypothetical scenarios were also constructed to predict the preventable childhood asthma disease burden under different air pollution control levels. The results showed that in 2019, air pollution caused 264,800–467,100 childhood asthma exacerbation cases and 622,800–1115,000 incident asthma cases among children, accounting for 7.1% − 12.5% and 31.4% − 56.2% of the total asthma children visits and incidence of childhood asthma in that year. The pollutant that has the greatest impact on childhood asthma is O_3_, followed by PM_2.5_ and NO_2_. The health impacts of the three pollutants were spatially distributed to be higher in the central and southern regions of China, and lower in the southwestern, northeastern, and northwestern regions. Chongqing was the city most affected by three types of pollutants. When pollutant concentrations comply with the WHO guidelines, up to 267,900 cases of childhood asthma exacerbations and 873,900 new-onset childhood asthma cases could be averted.

## 1. Introduction

Asthma stands as the fourth most prevalent chronic condition worldwide among children aged 5–14, accounting for elevated emergency department visits, hospitalization rates, and school absenteeism in pediatric populations [1]. In recent decades, the incidence of childhood asthma has shown an upward trend worldwide, particularly in low -income and middle-income countries [2]. Epidemiological surveys of childhood asthma in Chinese cities show that the prevalence of asthma among children aged 0–14 increased from 0.91% in 1990 to 3.02% in 2010 [3], with the incidence of childhood asthma increasing sharply by 50% every decade [4]. Research demonstrates air pollution contributes to pediatric asthma exacerbations and onset, with global estimates attributing 8%−20% (9–23 million cases) and 4%−9% (5–10 million cases) of annual childhood asthma emergency visits to ozone and PM₂.₅ exposure respectively [5]. Another study on the global burden of childhood asthma found that approximately 4 million asthma cases each year can be attributed to NO₂, with up to 48% of the urban childhood asthma burden attributable to NO₂ [6]. Studies on children in the United States have found that 18% to 42% of the childhood asthma burden can be attributed to air pollution (PM_10_, PM_2.5_, NO_2_) [7]. Using a mouse model, a toxicological study has shown a link between NO₂ exposure and an elevated risk of allergic asthma [8]. Several studies in China have also shown that exposure to air pollution is associated with childhood asthma, while persisting academic debate surrounds the etiological contributions of specific pollutants [9,10].

It can be seen that it is highly necessary to assess the impact of air pollution on childhood asthma. However, the contributions of different air pollutants to the health impacts of childhood asthma may vary due to factors such as region and climate. Furthermore, methodological gaps persist in health impact research, particularly concerning comprehensive epidemiologic investigations targeting Chinese pediatric populations. Therefore, this study aims to quantify the health impacts of childhood asthma caused by air pollution in China at the urban scale, analyze the contribution levels of air pollutants, and provide data support for effectively reducing the health impacts of childhood asthma. This study concurrently examines the health effects of PM₂.₅, NO₂, and O₃ on childhood asthma. This selection was motivated by two key reasons. First, there is substantial and significant evidence linking three pollutants to childhood asthma [5–7]. Furthermore, the influences of other pollutants, such as SO₂ and CO, on childhood asthma remain contentious, and there is a scarcity of relevant epidemiological studies conducted in China. A meta-analysis investigating multiple pollutants and asthma exacerbations found no association between CO and childhood asthma [11]. Additionally, a recent retrospective cohort study on childhood asthma in China identified a significant association with NO₂ but observed no link with SO₂ [12]. These considerations collectively precluded their inclusion in our current analysis.

## 2. Materials and methods

Taking cities as the basic unit, this paper estimates the health impact and economic loss of childhood asthma attributable to air pollution in 336 cities in China in 2019. The estimation results do not include Macao, Hong Kong, Taiwan, and Sansha. Since asthma is a chronic lifelong disease, the health effects of asthma are divided into short-term impact and long-term impact. The short-term impact refers to the childhood asthma exacerbation, while the long-term impact refers to new-onset childhood asthma.

### 2.1. Health impact estimation model

Many cases of asthma exacerbations can be self-treated with medication, but the health impacts of such cases are difficult to quantify. The third epidemiological survey of childhood asthma in Chinese cities indicated that within the most recent 1 year prior to the survey, 61.4% of children with asthma experienced wheezing attacks, among whom approximately 78.6% had a history of emergency department visits or hospitalizations [3]. Therefore, this paper defines emergency department visits and hospitalizations as the short-term impacts of air pollution on asthma exacerbation, and quantifies the health impacts on children with pre-existing asthma attributable to air pollution through the number of clinic visits due to asthma attacks. The formula is as follows:


$${\Delta Y}_{i}={POP}_{i}*{PREV}_{i}*BI*\left(\text{1}-e^{-\beta \Delta C}\right)$$ (1)

Where ΔY is the health impacts of hospital visits for childhood asthma exacerbations due to air pollution in city i; ${POP}_{i}$ is the population number of 0–14 age children in city i, ${PREV}_{i}$ is asthma prevalence in 0–14 age children in city i, BI is the baseline incidence of hospital visits for childhood asthma exacerbations (outpatient and emergency room, hospitalization, etc.) in the last year, β is the concentration-response factor of pollutants for city i (per 1 μg/m^3^), and ∆C is the change of pollutant concentration in city i in 2019. For the threshold concentration of the health effects of the pollutants, referring to several research achievements in China [13,14], it is assumed that there is no threshold concentration for the short-term effects of PM_2.5_, NO_2_, and O_3_.

For the estimation of new-onset asthma cases attributable to air pollution, we first multiply the number of at-risk children in the current year (children who have never asthma and children with newly diagnosed asthma cases) by the incidence rate, and then estimate the asthma incident cases caused by air pollution exposure by the population attributable fraction (PAF). The formula is as follows:


$${\Delta Asthma}_{i}=\left({POP}_{i}-{POP}_{i,\text{2}\text{0}\text{1}\text{8}}*{PREV}_{i,\text{2}\text{0}\text{1}\text{8}}\right)*{INC}_{i}*\left(\text{1}-e^{-\beta \Delta C}\right)$$ (2)

Where ${\Delta Asthma}_{i}$ is the number of new cases attributed to air pollution in city i, ${INC}_{i}$ is the incidence of asthma in 0–14 age children in city i. Different from the estimation of disease burden of asthma exacerbation, exposure levels above natural background concentrations were used in estimating asthma incident cases attributable to air pollution, due to the long-term impacts [11]. We use the lowest annual concentration of the city in 2019 as the background concentration.

### 2.2. Exposure-response coefficient from a systematic review

In order to increase the accuracy of estimation, the results of epidemiological studies in China were adopted for the relevant concentration-response coefficients. As there is no Meta-analysis on the relationship between air pollution and childhood asthma in China, this paper systematically searched the epidemiological research between air pollution and childhood asthma in China based on Web of science, CNKI, Wanfang database. The retrieval time is from the establishment of the database to December 31, 2020. The combination of Chinese and English keywords such as “children”, “childhood”, “asthma”, “air pollution/PM_2.5_/NO_2_/O_3_”, “China/Chinese” were used.

In June 2021, three researchers separately searched the Web of Science, CNKI, and Wanfang databases. After completing the searches, three researchers independently reviewed the titles and abstracts of all records and discussed any discrepancies until consensus was reached. Subsequently, two researchers independently reviewed the full-text articles selected for inclusion. Any disagreements were resolved through discussion to decide on inclusion or exclusion. If uncertainty persisted, a third researcher was consulted.

#### 2.2.1. Inclusion and exclusion criteria.

Inclusion criteria: The study population is Chinese children aged 0–14 years; The literatures are Published epidemiological/observational studies, including time-series, case-crossover, case-control, cross-sectional, and cohort studies; Asthma is explicitly specified as the outcome of the study; Air pollutants must include one or more of PM_2.5_, NO_2_ and O_3_, and the study results of single pollutant model are available; Time-series and case-crossover studies report quantitative associations between pollutants and asthma emergency room visits, outpatient visits, or hospitalizations; Case-control, cross-sectional, and cohort studies report quantitative associations between pollutants and children diagnosed with asthma by doctors.

Exclusion criteria: Non-original research, such as review and comment; Study on indoor air pollution exposure; the annual pollutant concentration is not provided; Repeated reports or studies using the same data; the literature of the fetus in the womb.

#### 2.2.2. Data extraction and processing.

Based on the research requirements of this study, we designed a data extraction form. Key information included in the form comprised: pollutant type, pollutant concentration, health outcome, children’s age, risk estimate and 95% confidence interval (95%CI), study design. Two researchers used this form to independently extract data from the finally selected articles. The extracted data were then compared, and any discrepancies were resolved. When necessary information was unclear, we contacted the authors of the articles to request additional details.

Among the three types of pollutants, PM_2.5_ and NO_2_ are generally measured by the daily average concentration, while the measurement method of O_3_ varies in different literatures, mainly including the daily maximum 1h concentration (O_3-M1h_), the daily maximum 8-hour average concentration (O_3-M8h_) and the daily average concentration (O_3-24h_). In order to make the study comparable, we use the conversion ratio standard (O_3-M1h_: O_3-M8h_: O_3-24h_ = 2:1.5:1) given by the United States EPA to convert all risk estimates and their 95% CI into standard estimates under the O_3-M8h_ [15].

For the convenience of analysis, the risk estimates (relative risk (RR), odds ratio (OR), and excess risk (ER)) given in various literatures are adjusted to the standardized RR value, that is, the RR (95%CI) for every 10 μg/m^3^ increase in pollutant concentration, as given by:


OR=RR (3)

RR=1+ER (4)

$${RR}_{\text{standardized}}={RR}_{\text{origin}}^{\text{increment}(\text{10})/\text{incremnte}(\text{origin})}$$ (5)

#### 2.2.3. Quality assessment.

Using the Newcastle–Ottawa Quality Assessment Scale (NOS), we evaluated each study’s validity. The evaluation of case–control studies and cross-sectional studies included three aspects: selection method between case group and control group, comparability between case group and control group, and exposure assessment method. Additionally, the evaluation of cohort studies and time series studies included the selection of cohort, comparability, and result measurement.

Both NOS checklists are given in the form of 8 questions and are designed to help the assessor think about the validity of each study. Each question is answered by ‘yes’, ‘no’, and ‘can’t tell’, and studies were included in the meta-analysis if they obtained five or more ‘yes’ answers. All included papers were independently evaluated.

#### 2.2.4. Meta-analysis.

The random effect model is used to summarize the risk estimates within the scope of the study. Assuming that the included studies are random results of all possible outcomes, it can explain the within-study variance due to chance and sampling error and the between-study variance caused by heterogeneity. All analyses were run in Stata SE 14.0. All tests were two-sided, and p < 0.05 is required for statistical significance. Initially, heterogeneity was examined with the I^2^ statistic, with an I^2^ value exceeding 50% typically denoting considerable heterogeneity. Subsequently, in cases of substantial heterogeneity, subgroup analyses were employed to investigate its potential origins. Lastly, we identified publication bias and assessed the robustness of the estimated results.

### 2.3. Pollutant concentrations and baseline data

The pollutant concentration data came from the national urban air quality real-time publishing platform, which released hourly concentration data. PM_2.5_ and NO_2_ adopt daily 24h average concentration, while O_3_ adopted daily maximum 8-hour average concentration. Due to the unavailability of city-scale data on the population of 0–14 children, the population of children in each city was estimated by the proportion of children aged 0–14 in the province and the total city population. National scale prevalence and incidence data for childhood asthma were obtained from the Institute for Health Metrics and Evaluation (IHME). The baseline data of hospital visits for childhood asthma exacerbations was based on the latest China Children Homes Health study.

### 2.4. Scenario setting

Three hypothetical scenarios were designed to estimate the health benefits of improved air quality (Table 1). In the first scenario, the air pollution level of each city does not exceed China ambient air quality standard Level-2 (S1). In the second scenario, the air pollution level of each city does not exceed China Ambient Air Quality Standard Level-1(S2). In the third scenario, the air pollution level of each city does not exceed the WHO air quality guidelines (S3).

**Table 1:** Standard concentrations of pollutants in different scenarios (μg/m^3^).

Scenario	PM_2.5_	NO_2_	O_3_
S1	35	40	160
S2	15	40	100
S3	5	10	100

## 3. Results

### 3.1. Urban air pollutant concentrations

According to the evaluation requirements and data validity requirements of China ambient air quality standards and China technical regulation for ambient air quality assessment (on trial), real-time monitoring data of each city are processed, so as to obtain the annual average air pollutant concentrations of 336 cities.

In 2019, for PM_2.5_, the annual concentration in cities is 36.5 μg/m^3^, with a standard deviation of 14.2. The lowest concentration is 6.6 μg/ m^3^ in Ngari Prefecture, and the highest concentration is 110.4 μg/ m^3^ in Hotan Prefecture. It is noteworthy that no city has yet complied with the WHO Air Quality Guideline (5 μg/m^3^) for annual average PM₂.₅ levels. It was observed that 47.8% of cities and 61.3% of children (0–14 years old, same below) resided in areas exceeding the 35 μg/m^3^ annual PM₂.₅ concentration. At the lower threshold of 15 μg/m^3^, these proportions rose sharply to 95.5% and 99.1%, respectively.

For NO_2_, the annual concentration in cities were 26.3 μg/ m^3^, with a standard deviation of 9.4. The lowest concentration is 6.8 μg/m3 in Ngari Prefecture, and the highest concentration is 50.3 μg/ m^3^ in Tangshan. It was observed that 10.1% of cities and 14.2% of children resided in areas exceeding the 40 μg/m^3^ annual NO_2_ concentration. At the lower threshold of 10 μg/m^3^, these proportions rose sharply to 97.3% and 99.2%, respectively.

For O_3_, the annual concentration in cities were 146.4 μg/ m^3^, with a standard deviation of 27.1. The lowest concentration is 78.4 μg/ m^3^ in Qamdo, and the highest concentration is 209.8 μg/m3 in Liaocheng. It was observed that 28.2% of cities and 39.9% of children resided in areas exceeding the 160 μg/m^3^ annual O_3_ concentration. At the lower threshold of 100 μg/m^3^, these proportions rose sharply to 97.3% and 99.2%, respectively.

### 3.2. Results of meta-analysis

#### 3.2.1. Results of literature collation.

We obtained 761 records through keyword searches in the databases. After removing duplicates, 680 records remained. Following title and abstract screening, 86 articles were selected for full-text review. Finally, we included 18 articles for further analysis. Characteristics are shown in Table 2. Due to the small number of studies that specifically focused on children aged 0–14, studies that included people under 18 were included in the analysis. The 18 papers were published from 2005 to 2020, and were conducted from 1997 to 2018. These studies include 11 time-series studies, 1 case-crossover study and 5 cross-sectional and 1 cohort study. Nine studies reported risk estimates for 3 pollutants simultaneously, two studies reported risk estimates for NO_2_ and O_3_, and seven studies reported risk estimates for 1 kind of pollutants.

**Table 2:** Characteristics of included studies.

Study	Area	Period	Pollutants	Study Design	Outcomes	Age
Hwang et al. [16]	Taiwan	2001	O_3_	cross-sectional	prevalence	0-15
Lee et al. [17]	Hong Kong	1997-2002	PM_2.5_ NO_2_ O_3_	time-series	hospitalization	0-18
Ko et al. [18]	Hong Kong	2000-2005	PM_2.5_ NO_2_ O_3_	time-series	hospitalization	0-14
Mou et al. [19]	Shanghai	2009-2010	PM_2.5_ NO_2_ O_3_	time-series	outpatient and emergency hospital visits	3-14
Liu et al. [9]	Seven northeastern cities	2009	NO_2_ O_3_	cross-sectional	prevalence	6-13
Hua et al. [20]	Shanghai	2007-2012	PM_2.5_	time-series	outpatient and emergency hospital visits hospitalization	0-14
Ding et al. [21]	Chongqing	2013	PM_2.5_ NO_2_ O_3_	case-crossover	outpatient and emergency hospital visits hospitalization	0-18
Deng et al. [22]	Changsha	2011-2012	NO_2_	cross-sectional	prevalence	3-6
Liu et al. [23]	Shanghai	2011-2012	NO_2_	cross-sectional	prevalence	3-6
Wang et al. [24]	Taipei	2010	PM_2.5_ NO_2_ O_3_	cohort	prevalence	3-6
Dai et al. [25]	Hong Kong	2005-2014	O_3_	time-series	hospitalization	0-18
Zhang et al. [26]	Hefei	2015-2016	PM_2.5_ NO_2_ O_3_	time-series	hospitalization	0-18
Kuo et al. [27]	Taiwan	2001-2012	PM_2.5_ NO_2_ O_3_	time-series	hospitalization	0-18
Norback et al. [28]	7 cities	2010-2012	NO_2_	cross-sectional	prevalence	3-6
Chang et al. [10]	Shenyang	2013-2017	PM_2.5_ NO_2_ O_3_	time-series	outpatient and emergency hospital visits	0-17
Liu et al. [29]	Shanghai	2016-2018	PM_2.5_ NO_2_ O_3_	time-series	emergency room visits	0-18
Hu et al. [30]	Shanghai	2007-2017	NO_2_ O_3_	time-series	outpatient and emergency hospital visits	0-18
Zhang et al. [13]	Hangzhou	2015-2017	PM_2.5_	time-series	Outpatient	0-14

For long-term impacts, Meta-analysis cannot be carried out because there are few relevant studies. Therefore, the data in the literature were directly used as the concentration-response factor of the incidence rate of childhood asthma, and the research results in multiple cities are mainly used. The RR values for childhood diagnosed-asthma are 1.14 (95%CI, 1.02,1.26) [24] and 1.19 (95%CI, 1.06,1.34) [28] and 1.12(95%CI, 1.09,1.16) [9] per 10 μg/m^3^ increase in PM_2.5_, NO_2_ and O_3_, respectively.

#### 3.2.2. Results of meta-analysis.

In order to make use of the existing epidemiological studies, the results of outpatient, outpatient and emergency, and hospitalization are uniformly classified into the types of hospital visits, so as to capture more cases of childhood asthma exacerbations attributable to air pollution. Most studies have reported a significant positive association between the concentration of pollutants and hospital visits for childhood asthma exacerbations. Meta-analysis results are shown in Table 3. The combined effect risks of all pollutants are statistically significant, indicating that air pollution will increase the risk of asthma exacerbation in children. NO_2_ has the greatest impact, a 10 μg/m^3^ increase in concentration is associated with 2.58% (95%CI,.49%, 3.68%) higher asthma hospital visits. The impact of PM_2.5_ ranks second, a 10 μg/m^3^ increase in concentration is associated with 2.10% (95%CI, 0.76%,3.46%) higher asthma hospital visits. The impact of O_3_ comes last, a 10 μg/m^3^ increase in concentration is associated with 0.87% (95%CI, 0.40%, 1.35%) higher asthma hospital visits. Sensitivity analysis showed that the results of meta-analysis for all pollutants were robust.

**Table 3:** Overall meta-analysis of hospital visits (per μg/m^3^).

Pollutant	RR(95%CI)		Estimated number	I^2^(%)/p	Egger p
	Overall	Trim and fill			
PM_2.5_	1.0210(1.0076,1.0346)*	1.002(0.990,1.015)	10	98.7/0.000	0.043
NO_2_	1.0258(1.0149,1.0368)*	1.013(1.003,1.024)*	9	95.1/0.000	0.021
O_3_	1.0087(1.0040,1.0135)*	1.000(0.995,1.006)	10	96.0/0.000	0.065

* p < 0.05.

Egger test showed that the meta-analysis of PM_2.5_ and NO_2_ in the three types of pollutants may have publication bias(p < 0.05). To address this, we further employed the trim-and-fill method to evaluate potential publication bias. The corresponding results of the random-effects model based on this analysis are also displayed in Table 3. Our results show that the RR estimates were not substantially altered by the trim-and-fill procedure. This observation indicates that the findings of our meta-analysis are robust.

#### 3.2.3. Heterogeneity analysis.

In general, the meta-analysis of three types of pollutants has high heterogeneity. To explore potential sources of heterogeneity, this paper conducted subgroup analyses. Due to the limited number of studies available for the Meta-analysis, these analyses were restricted to lag patterns (single-day lags and multi-day lags) and types of healthcare visits (hospital admissions and outpatient/emergency). A summary of all subgroup analysis results is provided in Table 4.

**Table 4:** Subgroup analyses (per μg/m^3^).

Pollutant	Subgroup	RR(95%CI)	I2(%)/p
PM_2.5_	Single-day lags	1.0104(1.0013,1.0196)*	96.4/0.000
	Multi-day lags	1.0213(1.0086,1.0342)*	71.9/0.014
	Hospital admissions	1.0393(1.0198,1.0592)*	92.6/0.000
	Outpatient/ emergency	1.0038(0.9993,1.0084)	76.1/0.002
NO_2_	Single-day lags	1.0171(0.9998,1.0347)	92.8/0.000
	Multi-day lags	1.0614(1.0289,1.0949)*	85.9/0.000
	Hospital admissions	1.0376(1.0235,1.0519)*	85.9/0.000
	Outpatient/ emergency	1.0126(1.0046,1.0208)*	79.2/0.002
O_3_	Single-day lags	1.0105(0.9988,1.0224)	93.5/0.000
	Multi-day lags	1.0151(0.9926,1.0381)	97.7/0.000
	Hospital admissions	1.0128(1.0031,1.0225)*	96.6/0.000
	Outpatient/emergency	1.0076(1.0024,1.0128)*	89.0/0.000

Regarding lag patterns, the between-group heterogeneity decreased for PM₂.₅ and NO₂, particularly under the multi-day lag condition, although levels remained elevated. As for O₃, subgroup heterogeneity showed little variation. Moreover, under the multi-day lag scheme, the heterogeneity for O₃ exceeded the overall heterogeneity. Regarding types of healthcare visits, a greater reduction in heterogeneity was observed for outpatient and emergency visits across all three pollutants, although heterogeneity remained elevated.

The subgroup analyses indicate that lag pattern is likely a contributor to heterogeneity in PM_2.5_ and NO₂ associations, and that healthcare visit type is a potential source of heterogeneity for all three pollutants. The available data did not permit a more thorough exploration of the heterogeneity observed in this paper.

### 3.3. Health losses of childhood asthma attributable to air pollution

Since all concentration-response factors are the results of the single pollution model, and the interaction between pollutants is not considered, this paper does not simply add up the health effects caused by PM_2.5_, NO_2_ and O_3_, but considers the assessment results as independent estimates of the potential impacts of different atmospheric pollutants. As shown in Table 5, in 2019, Among 336 cities, 30.37, 26.48, and 46.71 ten thousand cases of asthma exacerbation are attributable to PM_2.5_, NO_2_ and O_3_, respectively, accounting for 8.1%, 7.1%, and 12.5% of the total number of children with asthma in that year. 68.95, 62.28 and 111.50 ten thousand new asthma cases of children are attributable to PM_2.5_, NO_2_ and O_3_, respectively, accounting for 34.7%, 31.4%, and 56.2% of the total number of new-onset childhood asthma in that year. It can be seen that in the health impacts on childhood asthma, the long-term effects account for the main part. Moreover, regardless of short-term or long-term effects, O₃ causes the greatest health impacts, while the health impacts caused by PM_2.5_ and NO₂ are comparable.

**Table 5:** Burden of childhood asthma.

Outcome	Pollutant	Attributable cases(95%CI) (*ten thousands)
Asthma exacerbation	PM_2.5_	30.37(11.39,48.29)
	NO_2_	26.48(15.63,36.96)
	O_3_	46.71(22.32,69.73)
New-onset asthma	PM_2.5_	68.95(15.21,105.62)
	NO_2_	62.28(23.89,91.98)
	O_3_	111.50(93.26,130.02)

#### 3.3.1. Short-term health losses.

**Fig 1:**
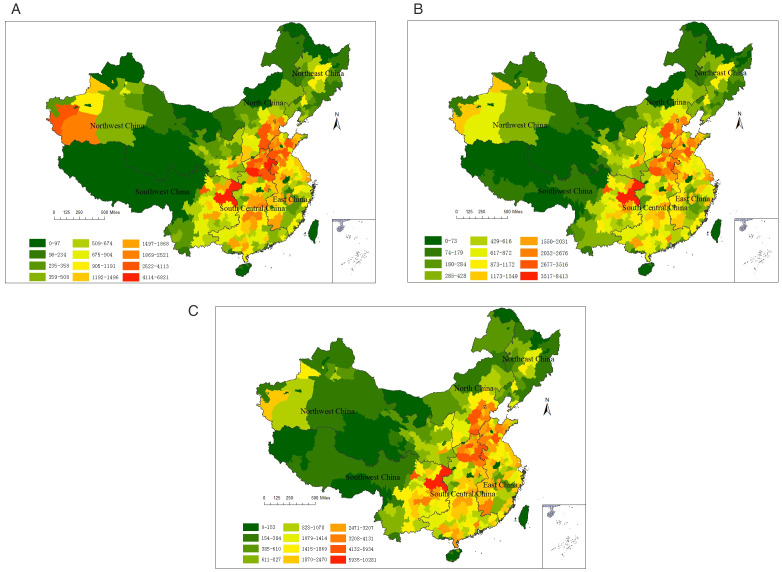
Spatial distribution of hospital visits cases for childhood asthma exacerbations attributable to air pollution. (**A**) PM_2.5_; (**B**) NO_2_; (**C**) O_3_.

Fig 1 shows the spatial distribution of hospital visits cases for childhood asthma exacerbations attributable to air pollution in 336 cities in 2019. The geographic data utilized in this study, specifically the administrative boundaries of prefecture-level cities in China, was sourced from the publicly accessible repository of the Resource and Environmental Science Data Platform [31]. Since the statistical results do not include Macao, Hong Kong, Taiwan, and Sansha, the values for these regions are defaulted to zero, and the same applies hereinafter. The short-term effects of the three pollutants all show similar spatial distributions, with higher values in South Central China and East China, and lower values in Southwest China, Northeast China, and Northwest China.

Regarding the short-term health impacts of PM_2.5_, the number of attributable hospital visits for childhood asthma exacerbations in each city ranged from 6 cases (in Ngari Prefecture) to 6,821 cases (in Chongqing). As shown in Table 6, the number of attributable cases in 35 cities did not exceed 100, mainly concentrated in provinces such as Tibet, Qinghai, Heilongjiang, Xinjiang, Gansu, and Yunnan. On the other hand, six cities recorded more than 4,000 attributable cases: Handan (4,002), Baoding (4,113), Zhoukou (4,630), Nanyang (4,774), Shangqiu (4,875), and Chongqing (6,821).

**Table 6:** The number of cities with different scales of short – term health losses.

Pollutant	0-100 cases	101-1000 cases	1001-4000 cases	4001-7000 cases	7001-11000 cases
PM2.5	35	193	102	6	0
NO2	33	213	89	0	1
O3	7	157	158	13	1

Regarding the short-term health impacts of NO₂, the number of attributable hospital visits for childhood asthma exacerbations in each city ranged from 13 cases (in Alxa League) to 8,413 cases (in Chongqing). Among them, 33 cities reported ≤100 attributable cases, primarily concentrated in Qinghai, Heilongjiang, Tibet, Xinjiang, and Yunnan provinces. On the other hand, 63% of cities reported attributable cases ranging between 101 and 1,000, while only Chongqing exceeded 4,000 cases.

Regarding the short-term health impacts of O_3_, the number of attributable hospital visits for childhood asthma exacerbations in each city ranged from 36 cases (in Ngari Prefecture) to 10281 cases (in Chongqing). Only seven cities reported ≤100 attributable cases: Ngari Prefecture (36 cases), Greater Khingan Mountains (60), Alxa League (61), Jiayuguan (64), Nyingchi (66), Golog Tibetan Autonomous Prefecture (71), and Huangnan Tibetan Autonomous Prefecture (82). Fourteen cities reported over 4,000 attributable cases, primarily concentrated in Henan, Hebei, Shandong provinces and Chongqing.

Spatial analysis revealed O₃ pollution as the dominant factor in short-term health impacts, with 51% of cities exceeding 1,000 attributable cases – significantly higher than PM₂.₅ (32%) and NO₂ (27%). Additionally, Chongqing was the most affected city by all three pollutants, while the least impacted areas were located in Tibet and Inner Mongolia. Geographical analysis (Table 7) revealed O₃ as the predominant contributor to childhood asthma exacerbations across all regions, accounting for 10.6%−14.7% of annual childhood asthma exacerbations visits. PM₂.₅-attributable cases represented 6.1%−9.7% of regional visits, while NO₂-related exacerbations comprised 6.1%−9.0%. North China is the region most affected by the short-term health impacts of PM_2.5_, NO_2_, and O_3_.

**Table 7:** Attributable cases of childhood asthma by region (10,000 cases).

Outcome	Pollutant	North China	Northeast China	East China	South Central China	Southwest China	Northwest China
Asthma exacerbation	PM_2.5_	3.90	1.22	8.73	10.12	3.84	2.55
	Proportion^a^	9.7%	7.2%	8.4%	8.5%	6.1%	8.8%
	NO_2_	3.64	1.05	7.86	7.86	3.82	2.24
	Proportion^a^	9.0%	6.2%	7.5%	6.6%	6.1%	7.7%
	O_3_	5.94	1.84	13.83	15.18	6.69	3.24
	Proportion^a^	14.7%	10.8%	13.2%	12.8%	10.6%	11.1%
New-onset asthma	PM_2.5_	8.86	2.81	19.99	22.97	8.64	5.67
	Proportion^b^	41.2%	31.1%	36.0%	36.3%	25.7%	36.5%
	NO_2_	8.74	2.44	18.74	18.36	8.69	5.32
	Proportion^b^	40.7%	27.0%	33.7%	29.0%	25.8%	34.3%
	O_3_	14.78	4.00	34.03	36.79	14.59	7.31
	Proportion^b^	68.8%	44.3%	61.3%	58.2%	43.4%	47.1%

^a^refers to the proportion of children with asthma exacerbation attributable to air pollution in the number of children with asthma exacerbation that year.

^b^refers to the proportion of new-onset childhood asthma attributable to air pollution in the number of new-onset childhood asthma that year.

#### 3.3.2. Long-term health losses.

**Fig 2:**
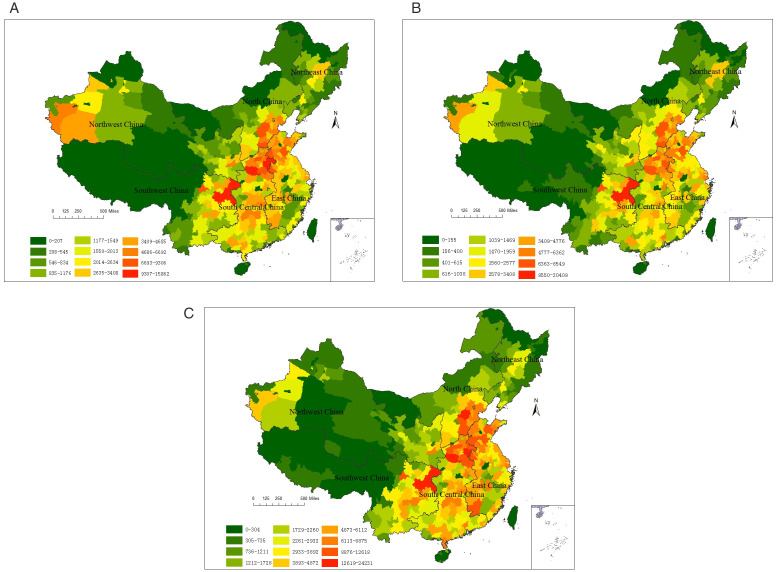
Spatial distribution of new-onset childhood asthma cases attributable to air pollution. **(A)** PM_2.5_; **(B)** NO_2_; **(C)** O_3_.

Fig 2 presented the spatial distribution of new-onset childhood asthma cases attributable to air pollution across 336 Chinese cities in 2019. The long-term health effects of all three pollutants exhibited spatial distributions similar to their short-term impacts, with elevated risks in South Central and East China, and lower levels in Southwest, Northeast, and Northwest China.

Regarding the long-term health impacts of PM_2.5_, the number of attributable new-onset childhood asthma cases in each city ranged from 0 cases (in Ngari Prefecture) to 15862 cases (in Chongqing). Among them, 36% and 55% of cities recorded between 0–1,000 and 1,001–5,000 attributable cases, respectively. Four cities reported over 10,000 attributable cases: Shangqiu (10,446), Zhoukou (10,502), Nanyang (10,753), and Chongqing (15,862). Regarding the long-term health impacts of NO2, the number of attributable new-onset childhood asthma cases in each city ranged from 0 cases (in Qamdo) to 24231 cases (in Chongqing). Among them, 40% and 52% of cities had attributable cases between 0–1,000 and 1,001–5,000 cases, respectively. Only Chongqing had more than 10,000 attributable cases. Regarding the long-term health impacts of O_3_, the number of attributable new-onset childhood asthma cases in each city ranged from 0 cases (in Nyingchi) to 20409 cases (in Chongqing). Among them, 21% and 59% of cities had attributable cases between 0–1,000 and 1,001–5,000 cases, respectively. Only Chongqing had more than 10,000 attributable cases. There were 14 cities with more than 10,000 attributable cases, mainly concentrated in Henan, Hebei, Shandong and Chongqing.

Spatial analysis revealed O₃ pollution as the dominant factor in long-term health impacts, with 21% of cities exceeding 5,000 attributable cases – significantly higher than PM₂.₅ (8%) and NO₂ (7%). Additionally, Chongqing was the most affected city by all three pollutants, while the least impacted areas were located in Tibet. From a geographical division perspective (Table 8), cases attributable to PM_2.5_, NO_2_, and O_3_ accounted for approximately 25.7%−41.2%, 25.8%−40.7%, and 43.4%−68.8% of new-onset childhood asthma cases in that year, respectively. North China remained the most affected region, while Southwest China was the least affected.

**Table 8:** The number of cities with different scales of long – term health losses.

Pollutant	0-1000 cases	1001-5000 cases	5001-10000 cases	10001-15000 cases	15001-20000 cases	20001-25000 cases
PM_2.5_	122	186	24	3	1	0
NO_2_	136	175	24	0	0	1
O_3_	70	197	55	13	0	1

### 3.4. Scenario analysis

This paper estimated the reducible health impacts of childhood asthma under different hypothetical scenarios, as shown in Table 9. Under the S1 scenario (where PM₂.₅, NO₂, and O₃ concentrations are reduced to China’s Grade II Ambient Air Quality Standards [35 μg/m^3^, 40 μg/m^3^, and 160 μg/m^3^, respectively]), projected reductions in childhood asthma exacerbations would reach 68,600, 4,000, and 28,100 cases, accounting for approximately 1.8%, 0.1%, and 0.8% of annual childhood asthma exacerbations cases, respectively. The preventable new-onset childhood asthma cases were projected to be 201,100 14,000 and 169,500 cases for PM₂.₅, NO₂, and O₃ under the S1 scenario, accounting for approximately 10.1%, 0.7%, and 8.5% of annual new-onset childhood asthma cases, respectively.

**Table 9:** Avoidable health burden under different scenarios.

Scenario	Outcome	Pollutant	Avoidable health burden/ Proportion^a^ (10,000 cases/%)
S1	Asthma exacerbation	PM_2.5_	6.86/1.8
		NO_2_	0.40/0.1
		O_3_	2.81/0.8
	New-onset asthma	PM_2.5_	20.11/10.1
		NO_2_	1.40/0.7
		O_3_	16.95/8.5
S2	Asthma exacerbation	PM_2.5_	19.54/5.2
		NO_2_	0.40/0.1
		O_3_	17.21/4.6
	New-onset asthma	PM_2.5_	54.27/27.3
		NO_2_	1.40/0.7
		O_3_	87.39/44.0
S3	Asthma exacerbation	PM_2.5_	26.79/7.2
		NO_2_	17.54/4.7
		O_3_	17.21/4.6
	New-onset asthma	PM_2.5_	71.59/36.1
		NO_2_	54.44/27.4
		O_3_	87.39/44.0

^a^represents the proportion of avoidable cases under hypothetical scenarios to total childhood asthma cases.

In the three hypothetical scenarios, the NO₂ standard is set at 40 μg/m^3^ for both S1 and S2, whereas the O₃ standard is set at 100 μg/m^3^ for S2 and S3. Our analysis shows negligible health benefits for childhood asthma from NO₂ attainment in scenarios S1 and S2, in terms of both short-term and long-term effects. This result is driven by two factors: firstly, 89.9% of Chinese cities already have NO₂ concentrations below the 40 μg/m^3^ threshold; secondly, the maximum urban concentration is only 10.3 μg/m^3^ above this standard, indicating a very small population exposed to excessive levels. In scenario S3, health benefits from NO₂ reduction begin to emerge for childhood asthma; nevertheless, NO₂ remains a secondary contributor rather than the maximal contributor to overall health gains.

Regarding short-term health effects, achieving the standard PM₂.₅ concentration level shows the greatest potential for reducing childhood asthma exacerbations. This outcome is attributed to two key factors: firstly, the child population exposed to PM₂.₅ concentrations above the standard in 2019 was the largest among all pollutants considered, independent of the threshold chosen. Furthermore, the short-term exposure-response coefficient for PM₂.₅ ranks second, differing only marginally from that of NO₂.

The analysis of long-term health effects reveals that, with the exception of scenario S1, attaining the O₃ standard concentration represents the optimal strategy, as it yields the most significant benefits in reducing childhood asthma prevalence. The maximal long-term health benefit observed upon O₃ attainment can be largely attributed to the significant variability (large standard deviation) in its ambient concentrations across urban China, which presents a greater scope for reduction. This is the decisive factor, whereas the long-term exposure-response coefficient and the exposed population size for O₃ are quite similar to those of PM₂.₅. It is evident that for the disease burden of childhood asthma, short-term health benefits can be most effectively increased by targeting PM₂.₅ pollution control, whereas long-term health gains would be best achieved by addressing O₃ pollution.

## 4. Discussion

### 4.1. Comparison of exposure-response coefficients for short-term impacts of air pollution

When estimating the health impact of air pollution on a national scale, the concentration-response factors derived from Meta-analysis are more appropriate. Meta-analysis can overcome the estimation uncertainty caused by a single study, and better resolve the heterogeneity among different regions and populations. This paper searched and compared the Meta-analysis literatures published in recent years on the relationship between air pollution and childhood asthma exacerbation (Table 10).

**Table 10:** Other meta-analysis of childhood asthma exacerbations (per 10 μg/m^3^).

Study	Study characteristics	Pollutant	RR (95%CI)
Zheng et al.[14]	Short-term, global, Emergency visits and hospitalization, time-series and case-crossover	O_3_	1.009(1.002,1.017)
		NO_2_	1.020(1.011,1.029)
		SO_2_	1.021(1.011,1.032)
Orellano, et al. [11]	Short-term, global, Emergency visits and hospitalization, case-crossover	NO_2_	1.040(1.001,1.060)^a^
		SO_2_	1.047(1.009,1.086)^a^
		PM_2.5_	1.022(1.000,1.045)
Zhang et al. [15]	Short-term, east Asia, hospitalization, time-series and case-crossover	SO_2_	1.057(1.008,1.108)
		NO_2_	1.035(1.025,1.046)
		CO	1.141(1.093,1.191)^b^
		O3	1.029(1.022,1.037)
		PM_10_	1.021(1.017,1.024)
		PM_2.5_	1.022(1.019,1026)
Zheng et al. [32]	Short-term, global, Emergency visits and hospitalization, time-series and case-crossover	O_3_	1.008 (1.005, 1.012)
		CO	1.018 (1.013, 1.023)^b^
		NO_2_	1.018 (1.013, 1.023)
		SO_2_	1.016 (1.011, 1.022)
		PM_10_	1.013 (1.008, 1.018)
		PM_2.5_	1.025 (1.013, 1.037)

a. represents per 10 ppb;

b. represents per 1 mg/m^3^.

Most studies define the effects of asthma exacerbations as emergency visits or hospital admissions and do not distinguish each other. Zheng et al. [14] systematically reviewed the effects of O_3_, NO_2_, and SO_2_ globally. Meta-analysis results showed that all three pollutants were associated with an increased risk of asthma exacerbation, and the association strength was higher in children than in adults. Orellano et al. [11] reviewed the short-term effects of PM_10_, PM_2.5_, NO_2_, SO_2_ and O_3_ on a global scale and found that only PM_10_ and SO_2_ did not show significant correlation with asthma exacerbation, and NO_2_, SO_2_ and PM_2.5_ were all significantly correlated with asthma exacerbation in the children subgroup. Zhang et al. [15] reviewed the short-term impact of air pollution in East Asia, and found that in the subgroup of children, the correlation between all pollutants and asthma hospitalization was statistically significant and stronger than that of the general population, especially for NO_2_, CO and PM_10_. Zheng et al. [32] found that six major air pollutants were statistically significant associated with asthma exacerbation, and had a stronger association with children. Previous meta-analysis studies have shown that childhood asthma exacerbations are more susceptible to the impact of air pollution. Except for one study [32], other studies have shown that the influence of NO_2_ is greater than that of PM_2.5_ and O_3_, which is consistent with the research results of this paper.

### 4.2. Uncertainty analysis

First, there are limitations regarding the availability of baseline data. As of October 2025, the most recent nationwide cross-sectional study on childhood asthma prevalence in China remains the third survey, which was conducted from September 2009 to August 2010. It should be noted that the childhood asthma consultation rate employed in our analysis is drawn directly from this survey and has not been updated or adjusted; therefore, a discrepancy with the actual 2019 situation should be considered. It was found in the two national surveys conducted in 2000 and 2010 that the rates of childhood asthma exacerbations and asthma-related hospitalizations both decreased [3]. It can be inferred that the synergistic influence of policy directives, technological progress in healthcare, deepened patient education, and environmental enhancements is contributing to a potential decline in the rate of hospital visits among the childhood asthmatic population. Therefore, the short-term effects of air pollution estimated in this study may represent an overestimation.

Second, it is important to acknowledge that our Meta-analysis relied on studies using single-pollutant models, and thus did not consider interactions among different pollutants. The issue of collinearity among different pollutants may lead to an overestimation of the health effects of air pollution. Upon developing separate multi-pollutant models for NO₂ and O₃, Ko et al. [18] reported that a positive association with asthma hospitalizations persisted only for O₃. The research results of ZHANG et al. [26] showed that no significant association between PM_2.5_ and childhood asthma hospitalizations was observed in the multi-pollutant model, indicating that the impact of PM_2.5_ in the single-pollutant model may be confounded by other air pollutants (such as NO_2_). Lee et al. [17] reported that, following adjustment for multi-pollutant effects, childhood asthma hospitalizations remained significantly associated with NO₂, PM₁₀, PM₂.₅, and O₃, supporting the existence of independent effects for each pollutant. In a two-pollutant model, Liu et al. [23] reported that the association of NO₂ with childhood asthma emergency visits remained significant, while the effects of PM₂.₅ and SO₂ were reduced and lost significance after including NO₂. Several China-specific studies found that single-pollutant models yielded higher estimates than multi-pollutant models [17,18,23,26], pointing to a likely overestimation in our results. It is essential that future studies incorporate multi-pollutant models to advance the accuracy of epidemiological estimates in this field. Additionally, during the literature screening process, only half of the titles and abstracts underwent double-screening, while the remaining records were screened only once. This approach may introduce some risk of error, but we believe it is unlikely to alter the overall results of the Meta-analysis.

Third, the impact of O3 on childhood asthma remains uncertain. Some scholars had not found a positive correlation between O_3_ concentration and childhood asthma attacks [21,27]. A cohort study in Taipei showed that O_3_ had a protective effect on childhood asthma [24]. Kuo et al. [27] reported in their multi-pollutant model that O₃ was inversely associated with childhood asthma hospitalizations.

## 5. Conclusions

This paper systematically reviewed the published relevant epidemiological studies. Meta-analyses showed that short-term exposures to PM_2.5_, NO_2_, and O_3_ were all significantly associated with an increased risk of childhood asthma exacerbations in China. Among them, NO_2_ had the greatest impact, while O_3_ had the least impact. Health impact assessment showed that 7.1%−12.5% of childhood asthma exacerbations and 31.4%−56.2% of new-onset childhood asthma in 336 cities across China in 2019 were attributable to air pollution. The estimation results varied depending on the selection of air pollutants. Both in short-term and long-term impacts, O_3_ was the pollutant that contributed the most to health effects, followed by PM_2.5_, and NO_2_. The health impacts of the three pollutants showed a spatial distribution pattern of relatively higher levels in Central South and East China, and relatively lower levels in Southwest, Northeast, and Northwest China. Chongqing was the city most affected by air pollutants.

In terms of the health impacts of air pollution in geographical regions, North China was the most affected region, approximately 9.0%−14.7% of childhood asthma exacerbations cases and 40.7%−68.8% of new-onset childhood asthma cases can be attributed to air pollution. The Southwest region was the least affected, with approximately 6.1%−10.6% of childhood asthma exacerbations cases and 25.7%−43.4% of new-onset childhood asthma cases attributable to air pollution.

In the hypothetical scenarios, when air quality meets the second-level standard of China Ambient Air Quality Standards, reducing PM_2.5_ concentrations can maximize the prevention of childhood asthma. When air quality meets the first-level standard of China Ambient Air Quality Standards and the WHO guideline values, reducing PM_2.5_ concentrations can maximize the prevention of childhood asthma exacerbations, while reducing O_3_ concentration can maximize the prevention of new-onset childhood asthma. When the pollutant concentration levels reach the WHO guideline values, up to 267,900 childhood asthma exacerbations cases and 873,900 new-onset childhood asthma cases can be reduced.

## Supporting information

S1 Table Ambient pollutant concentrations and the associated burden of childhood asthma of 336 cities(excel). (XLSX)

## References

[1] GBD 2015 Chronic Respiratory Disease Collaborators. Global, regional, and national deaths, prevalence, disability-adjusted life years, and years lived with disability for chronic obstructive pulmonary disease and asthma, 1990-2015: a systematic analysis for the Global Burden of Disease Study 2015. Lancet Respir Med. 2017;5(9):691–706. doi: 10.1016/S2213-2600(17)30293-X 28822787 PMC5573769

[2] Baïz N, Annesi-Maesano I. Is the asthma epidemic still ascending?. Clin Chest Med. 2012;33(3):419–29. doi: 10.1016/j.ccm.2012.06.001 22929092

[3] National Cooperative Group on Childhood Asthma, Institute of Environmental Health and Related Product Safety, Chinese Center for Disease Control and Prevention, Chinese Center for Disease Control and Prevention. Third nationwide survey of childhood asthma in urban areas of China. Zhonghua Er Ke Za Zhi. 2013;51(10):729–35. 24406223

[4] Zhou X, Hong J. Pediatric Asthma Management in China: Current and Future Challenges. Paediatr Drugs. 2018;20(2):105–10. doi: 10.1007/s40272-017-0276-7 29222627

[5] Anenberg SC, Henze DK, Tinney V, Kinney PL, Raich W, Fann N, et al. Estimates of the Global Burden of Ambient PM2.5, Ozone, and NO2 on Asthma Incidence and Emergency Room Visits. Environ Health Perspect. 2018;126(10):107004. doi: 10.1289/EHP3766 30392403 PMC6371661

[6] Achakulwisut P, Brauer M, Hystad P, Anenberg SC. Global, national, and urban burdens of paediatric asthma incidence attributable to ambient NO2 pollution: estimates from global datasets. Lancet Planet Health. 2019;3(4):e166–78. doi: 10.1016/S2542-5196(19)30046-4 30981709

[7] Alotaibi R, Bechle M, Marshall JD, Ramani T, Zietsman J, Nieuwenhuijsen MJ, et al. Traffic related air pollution and the burden of childhood asthma in the contiguous United States in 2000 and 2010. Environ Int. 2019;127:858–67. doi: 10.1016/j.envint.2019.03.041 30954275

[8] Lu C, Wang F, Liu Q, Deng M, Yang X, Ma P. Effect of NO2 exposure on airway inflammation and oxidative stress in asthmatic mice. J Hazard Mater. 2023;457:131787. doi: 10.1016/j.jhazmat.2023.131787 37295329

[9] Liu F, Zhao Y, Liu Y-Q, Liu Y, Sun J, Huang M-M, et al. Asthma and asthma related symptoms in 23,326 Chinese children in relation to indoor and outdoor environmental factors: the Seven Northeastern Cities (SNEC) Study. Sci Total Environ. 2014;497–498:10–7. doi: 10.1016/j.scitotenv.2014.07.096 25112820

[10] Chang Q, Liu S, Chen Z, Zu B, Zhang H. Association between air pollutants and outpatient and emergency hospital visits for childhood asthma in Shenyang city of China. Int J Biometeorol. 2020;64(9):1539–48. doi: 10.1007/s00484-020-01934-9 32388688

[11] Orellano P, Quaranta N, Reynoso J, Balbi B, Vasquez J. Effect of outdoor air pollution on asthma exacerbations in children and adults: Systematic review and multilevel meta-analysis. PLoS One. 2017;12(3):e0174050. doi: 10.1371/journal.pone.0174050 28319180 PMC5358780

[12] Lu C, Zhang Y, Li B, Zhao Z, Huang C, Zhang X, et al. Interaction effect of prenatal and postnatal exposure to ambient air pollution and temperature on childhood asthma. Environ Int. 2022;167:107456. doi: 10.1016/j.envint.2022.107456 35952466

[13] Zhang W, Yu X, Ye C, Ye H, Xu X. The Impact of Atmospheric PM2.5 on Daily Outpatient Visits of Children for Asthma in Hangzhou in 2015-2017. Chinese Preventive Medicine. 2020;21(01):65–9. doi: 10.16506/j.1009-6639.2020.01.014

[14] Zheng X-Y, Orellano P, Lin H-L, Jiang M, Guan W-J. Short-term exposure to ozone, nitrogen dioxide, and sulphur dioxide and emergency department visits and hospital admissions due to asthma: A systematic review and meta-analysis. Environ Int. 2021;150:106435. doi: 10.1016/j.envint.2021.106435 33601224

[15] Zhang S, Li G, Tian L, Guo Q, Pan X. Short-term exposure to air pollution and morbidity of COPD and asthma in East Asian area: A systematic review and meta-analysis. Environ Res. 2016;148:15–23. doi: 10.1016/j.envres.2016.03.008 26995350

[16] Hwang B-F, Lee Y-L, Lin Y-C, Jaakkola JJK, Guo YL. Traffic related air pollution as a determinant of asthma among Taiwanese school children. Thorax. 2005;60(6):467–73. doi: 10.1136/thx.2004.033977 15923246 PMC1747433

[17] Lee SL, Wong WHS, Lau YL. Association between air pollution and asthma admission among children in Hong Kong. Clin Exp Allergy. 2006;36(9):1138–46. doi: 10.1111/j.1365-2222.2006.02555.x 16961713 PMC1618810

[18] Ko FWS, Tam W, Wong TW, Lai CKW, Wong GWK, Leung T-F, et al. Effects of air pollution on asthma hospitalization rates in different age groups in Hong Kong. Clin Exp Allergy. 2007;37(9):1312–9. doi: 10.1111/j.1365-2222.2007.02791.x 17845411

[19] Mou J, Peng L, Yang D, Jiang F, Yin Y, Hua J, et al. Influence of Weather and Pollution on the Number of Children with Asthma in Shanghai. Chinese Journal of Health Statistics. 2014;31(05):827–9.

[20] Hua J, Yin Y, Peng L, Du L, Geng F, Zhu L. Acute effects of black carbon and PM₂.₅ on children asthma admissions: a time-series study in a Chinese city. Sci Total Environ. 2014;481:433–8. doi: 10.1016/j.scitotenv.2014.02.070 24631605

[21] Ding L, Zhu D, Peng D, Zhao Y. Air pollution and asthma attacks in children: A case-crossover analysis in the city of Chongqing, China. Environ Pollut. 2017;220(Pt A):348–53. doi: 10.1016/j.envpol.2016.09.070 27692885

[22] Deng Q, Lu C, Ou C, Chen L, Yuan H. Preconceptional, prenatal and postnatal exposure to outdoor and indoor environmental factors on allergic diseases/symptoms in preschool children. Chemosphere. 2016;152:459–67. doi: 10.1016/j.chemosphere.2016.03.032 27003368

[23] Liu W, Huang C, Hu Y, Fu Q, Zou Z, Sun C, et al. Associations of gestational and early life exposures to ambient air pollution with childhood respiratory diseases in Shanghai, China: A retrospective cohort study. Environ Int. 2016;92–93:284–93. doi: 10.1016/j.envint.2016.04.019 27128713

[24] Wang I-J, Tung T-H, Tang C-S, Zhao Z-H. Allergens, air pollutants, and childhood allergic diseases. Int J Hyg Environ Health. 2016;219(1):66–71. doi: 10.1016/j.ijheh.2015.09.001 26404109

[25] Dai Y, Qiu H, Sun S, Yang Y, Lin H, Tian L. Age-dependent effect of ambient ozone on emergency asthma hospitalizations in Hong Kong. J Allergy Clin Immunol. 2018;141(4):1532-1534.e5. doi: 10.1016/j.jaci.2018.01.006 29382596

[26] Zhang Y, Ni H, Bai L, Cheng Q, Zhang H, Wang S, et al. The short-term association between air pollution and childhood asthma hospital admissions in urban areas of Hefei City in China: A time-series study. Environ Res. 2019;169:510–6. doi: 10.1016/j.envres.2018.11.043 30544078

[27] Kuo C-Y, Chan C-K, Wu C-Y, Phan D-V, Chan C-L. The Short-Term Effects of Ambient Air Pollutants on Childhood Asthma Hospitalization in Taiwan: A National Study. Int J Environ Res Public Health. 2019;16(2):203. doi: 10.3390/ijerph16020203 30642061 PMC6351918

[28] Norbäck D, Lu C, Zhang Y, Li B, Zhao Z, Huang C, et al. Sources of indoor particulate matter (PM) and outdoor air pollution in China in relation to asthma, wheeze, rhinitis and eczema among pre-school children: Synergistic effects between antibiotics use and PM10 and second hand smoke. Environ Int. 2019;125:252–60. doi: 10.1016/j.envint.2019.01.036 30731375

[29] Liu L, Liu C, Chen R, Zhou Y, Meng X, Hong J, et al. Associations of short-term exposure to air pollution and emergency department visits for pediatric asthma in Shanghai, China. Chemosphere. 2021;263:127856. doi: 10.1016/j.chemosphere.2020.127856 32822929

[30] Hu Y, Xu Z, Jiang F, Li S, Liu S, Wu M, et al. Relative impact of meteorological factors and air pollutants on childhood allergic diseases in Shanghai, China. Sci Total Environ. 2020;706:135975. doi: 10.1016/j.scitotenv.2019.135975 31841850

[31] Xu XL. A multi-year dataset of administrative boundaries for prefecture-level cities in China. Resource and Environmental Science Data Platform. 2023. doi: 10.12078/2023010102

[32] Zheng X, Ding H, Jiang L, Chen S, Zheng J, Qiu M, et al. Association between Air Pollutants and Asthma Emergency Room Visits and Hospital Admissions in Time Series Studies: A Systematic Review and Meta-Analysis. PLoS One. 2015;10(9):e0138146. doi: 10.1371/journal.pone.0138146 26382947 PMC4575194

